# Identification of approved drugs that inhibit the binding of amyloid β oligomers to ephrin type‐B receptor 2

**DOI:** 10.1002/2211-5463.12056

**Published:** 2016-04-01

**Authors:** Koichiro Suzuki, Takahiro Aimi, Tomoaki Ishihara, Tohru Mizushima

**Affiliations:** ^1^ Division of Drug Discovery and DevelopmentFaculty of PharmacyKeio UniversityMinato‐kuTokyoJapan; ^2^ Research Fellow of Japan Society for the Promotion of ScienceChiyoda‐kuTokyoJapan; ^3^ LTT Bio‐Pharma Co., LtdMinato‐kuTokyoJapan

**Keywords:** Alzheimer's disease, amyloid β oligomer, ephrin type‐B receptor 2

## Abstract

Ephrin type‐B receptor 2 (EphB2) is a member of the receptor tyrosine kinase family and plays an important role in learning and memory functions. In patients with Alzheimer's disease (AD) and in mouse models of AD, a reduction in the hippocampal EphB2 level is observed. It was recently reported that normalization of the EphB2 level in the dentate gyrus rescues memory function in a mouse model of AD, suggesting that drugs that restore EphB2 levels may be beneficial in the treatment of AD. Amyloid β (Aβ) oligomers, which are believed to be key molecules involved in the pathogenesis of AD, induce EphB2 degradation through their direct binding to EphB2. Thus, compounds that inhibit the binding of Aβ oligomers to EphB2 may be beneficial. Here, we screened for such compounds from drugs already approved for clinical use in humans. Utilizing a cell‐free screening assay, we determined that dihydroergotamine mesilate, bromocriptine mesilate, cepharanthine, and levonorgestrel inhibited the binding of Aβ oligomers to EphB2 but not to cellular prion protein, another endogenous receptor for Aβ oligomers. Additionally, these four compounds did not affect the binding between EphB2 and ephrinB2, an endogenous ligand for EphB2, suggesting that the compounds selectively inhibited the binding of Aβ oligomers to EphB2. This is the first identification of compounds that selectively inhibit the binding of Aβ oligomers to EphB2. These results suggest that these four compounds may be safe and effective drugs for treatment of AD.

**Abbreviations**
ABTS: 2,2′‐azino‐bis(3‐ethylbenzothiazoline‐6‐sulfonic acid)
AD: Alzheimer's disease
Aβ: amyloid β peptide
BRO: bromocriptine mesilate
BSA: bovine serum albumin
CEP: cepharanthine
DIH: dihydroergotamine mesilate
DMEM: Dulbecco's modified Eagle medium
DMSO: dimethyl sulfoxide
EphB2: ephrin type‐B receptor 2
HFIP: 1,1,1,3,3,3‐hexafluoro‐2 propanol
HRP: horseradish peroxidase
LCA: lithocholic acid
LEV: levonorgestrel
LTP: long‐term potentiation
PBS: phosphate‐buffered saline
PrP^C^: cellular prion protein
SD: standard deviation

Alzheimer's disease (AD) is a progressive neurodegenerative disease and the most common cause of dementia. Because the world's population is aging, the number of AD patients has been increasing; however, there are currently no disease‐modifying treatments for AD. Thus, development of disease‐modifying drugs is important.

One hallmark pathological feature of AD is senile plaques, which are composed of amyloid β peptide (Aβ). Monomeric Aβ easily self‐assembles to form soluble oligomers, protofibrils, and fibrils. A correlation between the amount of soluble Aβ oligomers and cognitive impairment in AD patients has been reported 1. In addition, soluble Aβ oligomers inhibit long‐term potentiation (LTP) 2, 3 which is involved in learning and memory functions 4. Therefore, Aβ oligomers are believed to play an important role in the learning and memory dysfunction observed in patients with AD.

Some endogenous receptors for Aβ oligomers, such as ephrin type‐B receptor 2 (EphB2) and cellular prion protein (PrP^C^), appear to be involved in the inhibitory effects of Aβ oligomers on LTP 5, 6, 7. EphB2, a member of the receptor tyrosine kinase family 8, is a receptor for ephrinB ligands (B1, B2, and B3) and ephrinA5 9 and functions in synaptic plasticity and synapse formation 10, 11, 12. Mice lacking EphB2 exhibit LTP impairment and memory dysfunction 11, 12. Reduced EphB2 levels are observed in postmortem hippocampal tissue from patients with AD 13 and in mouse models of AD 5, 13, 14. Aβ oligomers directly bind to EphB2 and induce its internalization and proteasome‐mediated degradation 5. In addition, artificial expression of EphB2 in the dentate gyrus rescues LTP and memory function in a mouse model of AD 5. These results suggest that inhibiting Aβ oligomer binding to EphB2 may be therapeutically beneficial in the treatment of AD.

The number of drugs reaching the marketplace each year is decreasing, mainly due to unexpected adverse effects being revealed at advanced stages of clinical trials. To overcome this problem, we took advantage of a recent strategy for drug discovery and development that focuses on examining drugs already approved for use in humans (i.e., drug repositioning). In this strategy, compounds with clinically beneficial pharmacological activity are screened from a library of approved drugs already in clinical use to be developed for new indications. One major advantage of this strategy is a decreased risk for unexpected adverse effects in humans because the safety of these drugs has already been well characterized in humans 15.

In the present study, from a library of approved drugs already in clinical use, we screened for compounds that inhibited the binding of Aβ oligomers to EphB2. We identified dihydroergotamine mesilate (DIH), bromocriptine mesilate (BRO), cepharanthine (CEP), and levonorgestrel (LEV). Although these compounds inhibited the binding of Aβ oligomers to EphB2, they did not inhibit the binding of an endogenous ligand, ephrinB2, to EphB2. This is the first time that compounds that selectively inhibited the binding of Aβ oligomers to EphB2 have been identified.

## Materials and methods

### Materials

Biotin‐conjugated Aβ42 (biotin‐Aβ) was obtained from AnaSpec Inc. (Fremont, CA, USA). EphB2 receptor ectodomains fused to the Fc portions of human IgG (EphB2‐Fc) and biotinylated ephrinB2‐Fc were purchased from R&D systems Inc. (Minneapolis, MN, USA). His‐tagged human PrP^C^ was obtained from Merck Co. (Darmstadt, Germany). Bovine serum albumin (BSA) and dimethyl sulfoxide (DMSO) were purchased from Sigma‐Aldrich Co. (St. Louis, MI). Horseradish peroxidase‐conjugated streptavidin (HRP‐streptavidin) was obtained from GE Healthcare UK Ltd. (Little Chalfont, UK). The 2,2′‐azino‐bis(3‐ethylbenzothiazoline‐6‐sulfonic acid) (ABTS) was obtained from Kirkegaard & Perry Laboratories, Inc. (Gaithersburg, MD, USA). The antibodies against Aβ (6E10) and PrP^C^ (ICSM35) were purchased from Covance Inc. (Emeryville, CA, USA) and D‐Gen Co. (London, UK), respectively. Phenol red‐free Dulbecco's modified Eagle medium (DMEM) and 1,1,1,3,3,3‐hexafluoro‐2 propanol (HFIP) were obtained from Wako Pure Chemical Industries Ltd. (Osaka, Japan). The library of drugs approved for clinical use was obtained from our laboratory stock 16.

### Aβ monomer and oligomer preparation

Aβ monomers and oligomers were prepared as described previously 16. Biotin‐Aβ was dissolved in HFIP and incubated at room temperature for 1 h and then on ice for 10 min. The HFIP was evaporated and the peptides were dissolved in DMSO for a final concentration of 10 mm. The peptides were diluted in phenol red‐free DMEM to give a final concentration of 100 μm and subjected to sonication (AIWA Co., Tokyo, Japan) for 10 min.

For oligomer preparation, the Aβ solution described above was incubated at 37 °C for 16 h and then centrifuged at 20 400 ***g*** for 15 min. The supernatant was frozen in liquid nitrogen and stored at −80 °C until use.

For monomer preparation, the sample was centrifuged and stored in a manner similar to that for oligomer preparation, without the incubation at 37 °C for 16 h.

### Characterization of Aβ oligomers by immunoblotting

To characterize Aβ oligomers, the supernatant was mixed with equal amounts of sample buffer and boiled at 98 °C for 5 min. Samples were applied to polyacrylamide sodium dodecyl sulfate gels and subjected to electrophoresis, after which proteins were immunoblotted with 6E10.

### Cell‐free binding assay

The cell‐free binding assay was performed as described previously 16. To evaluate binding between Aβ and EphB2, EphB2‐Fc was dissolved in phosphate‐buffered saline (PBS) to a final concentration of 0.5 μg·mL^−1^ and applied to a 96‐well ELISA plate and left at 4 °C overnight. Each well was washed with PBS containing 0.05% Tween 80 (T‐PBS) and blocked with 2% BSA at room temperature for 1 h. Then, each well was washed with T‐PBS and incubated with both biotin‐Aβ (200 nm) and each tested drug in DMEM at room temperature for 1 h. Each well was then washed with T‐PBS and incubated with HRP‐streptavidin in PBS (1 : 2000 dilution) at room temperature for 30 min. Each well was washed with T‐PBS and incubated with ABTS. Absorbance at 412 nm was measured using a plate reader (Infinite M1000; TECAN Group Ltd., Mannedorf, Switzerland). Biotin‐Aβ binding to EphB2 was calculated as follows:

OD_Blank_: absorbance of well without both EphB2 and each tested drug
OD_Control_: absorbance of well with EphB2 but without any tested drug
OD_Drug_: absorbance of well with both EphB2 and each tested drug

$$A\beta \;\mathrm{binding}\left({\text{OD}}_{412}\right)={\text{OD}}_{\mathrm{Control}}-{\text{OD}}_{\mathrm{Blank}}$$

$$A\beta \;\mathrm{binding}\left(\%\right)=\frac{{\text{OD}}_{\mathrm{Drug}}-{\text{OD}}_{\mathrm{Blank}}}{{\text{OD}}_{\mathrm{Control}}-{\text{OD}}_{\mathrm{Blank}}}\times 100$$

As a quality parameter for this assay, *Z*′‐factor 17 was calculated as follows:

μ_Control_: mean of OD_Control_
σ_Control_: standard deviation of OD_Control_
μ_Blank_: mean of OD_Blank_
σ_Blank_: standard deviation of OD_Blank_

$$Z^{\prime }=1-\frac{3\times \sigma_{\mathrm{Control}}+3\times \sigma_{\mathrm{Blank}}}{|\mu_{\mathrm{Control}}-\mu_{\mathrm{Blank}}|}$$

To evaluate binding between Aβ and PrP^C^, EphB2‐Fc was replaced with His‐tagged human PrP^C^.

To evaluate binding between ephrinB2 and EphB2, biotin‐Aβ in DMEM was replaced with biotinylated ephrinB2‐Fc in PBS (500 ng·mL^−1^).

### Chemical library and screening

Each drug in the library was dissolved in water or DMSO, depending on the drug. The concentration of each drug in the library was 1 mm, 5 mm, 10 mm, 25 mm, 50 mm, 100 mm, 2 mg·mL^−1^, 10 mg·mL^−1^, or 100 mg·mL^−1^, depending on the drug. Each drug was diluted 1000 times for the screening (final concentration, 1 μm, 5 μm, 10 μm, 25 μm, 50 μm, 100 μm, 2 μg·mL^−1^, 10 μg·mL^−1^, or 100 μg·mL^−1^). We confirmed that 0.1% of water or DMSO did not affect this assay. Screening was performed in duplicate wells per each drug in the same reader plate.

### Statistical analysis

All values are expressed as the mean ± standard deviation (SD). One‐way analysis of variance (anova) followed by Tukey's test was used to evaluate differences among three or more groups. Differences were considered statistically significant at *P* values less than 0.05.

## Results and Discussion

### Screening of approved drugs for compounds that inhibit the binding of Aβ oligomers to EphB2

**Figure 1:**
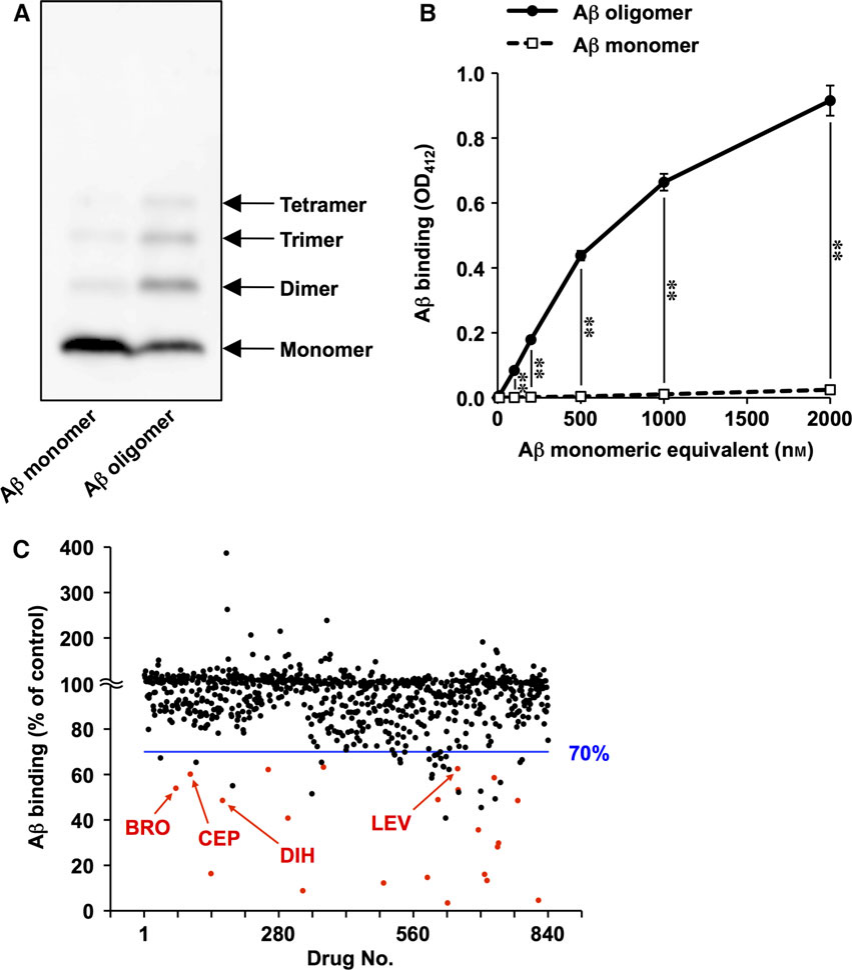
Screening of approved drugs for inhibitors of Aβ oligomer binding to EphB2. Aβ monomers and oligomers were prepared. (A) Both monomeric and oligomeric forms of biotin‐Aβ were analyzed by immunoblotting. (B) The abilities of the monomeric and oligomeric forms of biotin‐Aβ to bind to EphB2 were compared using a cell‐free binding assay. Values are mean ± SD (*n* = 3). **P < 0.01. (C) Each of 840 approved drugs was added simultaneously with Aβ oligomers to EphB2‐coated wells, and the amount of bound biotin‐Aβ was determined. Values are means (*n* = 2, duplicate wells per each drug in the same plate). Red dots indicate the 22 compounds that are listed in Table 1. Arrows indicate dihydroergotamine mesilate (DIH), bromocriptine mesilate (BRO), cepharanthine (CEP), and levonorgestrel (LEV).

To screen for inhibitors of Aβ oligomer binding to EphB2, we used a cell‐free binding assay. In this assay, biotin‐Aβ was applied to EphB2‐coated wells, and after washing, the amount of bound biotin‐Aβ was determined using HRP‐streptavidin. Aβ oligomers were prepared by incubation at 37 °C for 16 h, and we confirmed the formation of Aβ oligomers by immunoblotting (Fig. 1A).

We first compared the ability of Aβ monomers and oligomers to bind EphB2. We found that Aβ oligomers bound to EphB2 more efficiently than did the monomers (Fig. 1B). This result was consistent with a previous report 5 and suggested that this assay was appropriate for screening compounds that inhibited the binding of Aβ oligomers to EphB2.

From a library of 840 drugs approved for clinical use, we screened for compounds that inhibited the binding of Aβ oligomers to EphB2. Each compound was applied to the cell‐free binding assay simultaneously with Aβ oligomers, and the amount of bound biotin‐Aβ was determined (Fig. 1C). As an assay quality parameter, we calculated *Z*′‐factor to be 0.89, supporting the quality of this assay. Among these 840 compounds, 52 compounds inhibited more than 30% of the Aβ oligomer binding to EphB2. We examined the effect of these compounds on the binding in additional two independent experiments. We selected 22 compounds that inhibited more than 30% of the Aβ oligomer binding to EphB2 in both experiments (Table 1, red dots in Fig. 1C).

**Table 1:** Inhibitory effect of each compound on the binding of Aβ oligomers to EphB2 or PrP^C^

Compound	Aβ binding (% of control)			
	EphB2			
	Screening	Reproducibility		PrP^C^
Montelukast sodium	3.5	0.0	6.0 ± 0.4	0.4 ± 0.8
Pirarubicin hydrochloride	4.6	19.7 ± 1.8	33.1 ± 3.6	55.8 ± 9.3
Minocycline hydrochloride	8.8	3.9 ± 1.4	4.7 ± 2.1	4.2 ± 5.9
Tosufloxacin tosilate	12.2	11.2 ± 0.7	19.1 ± 2.6	42.2 ± 1.7
Diclazuril	13.3	11.3 ± 1.7	20.8 ± 5.1	12.4 ± 4.8
Suramin sodium	14.7	16.4 ± 1.9	21.2 ± 0.6	8.6 ± 4.0
Miltefosine	16.0	50.1 ± 1.9	53.0 ± 4.4	43.4 ± 2.3
Cytochrome *c*	16.4	27.1 ± 1.9	32.0 ± 2.0	53.4 ± 2.9
l‐thyroxine	28.1	53.8 ± 7.0	60.1 ± 3.7	61.0 ± 9.3
Abamectin	29.8	40.1 ± 2.1	45.4 ± 5.7	35.4 ± 12.5
Nystatin	35.6	33.2 ± 4.7	57.5 ± 0.8	41.6 ± 5.0
Lysozyme hydrochloride	40.8	48.7 ± 3.8	60.6 ± 2.0	48.3 ± 1.2
Bexarotene	48.5	32.7 ± 1.7	46.2 ± 1.2	23.3 ± 5.9
Dihydroergotamine mesilate	48.6	62.7 ± 6.7	65.3 ± 5.6	82.7 ± 5.8
Retinoic acid	48.9	39.5 ± 13.9	62.8 ± 2.3	27.8 ± 1.5
Mifepristone	53.3	49.1 ± 8.8	55.7 ± 2.2	20.7 ± 2.0
Bromocriptine mesilate	53.9	57.9 ± 6.3	69.5 ± 5.8	99.4 ± 9.3
Toltrazuril	58.6	55.8 ± 1.6	61.2 ± 8.7	35.9 ± 4.5
Cepharanthine	60.2	50.1 ± 4.0	66.3 ± 7.4	113.4 ± 4.5
Indigocarmine	62.2	55.9 ± 2.4	64.1 ± 8.5	47.4 ± 4.2
Levonorgestrel	62.6	64.1 ± 2.0	65.2 ± 4.2	95.6 ± 6.3
Oxytetracycline hydrochloride	63.2	53.7 ± 3.2	65.7 ± 4.8	58.0 ± 3.7

Among 52 compounds that inhibited more than 30% of the Aβ oligomer binding to EphB2 in the screening, 22 compounds that inhibited more than 30% of the Aβ oligomer binding to EphB2 in additional two independent experiments are listed. The effects of these 22 compounds on the binding of Aβ oligomers to EphB2 in the screening and in the additional two independent experiments (reproducibility) and on the binding of Aβ oligomers to PrP^C^ were shown. The four compounds that inhibited less than 30% of the Aβ oligomer binding to PrP^C^ are highlighted in gray. Values are mean (*n* = 2, ‘Screening’ column) or mean ± SD (*n* = 3). The concentration of nystatin or retinoic acid was 50 μm in the screening or 100 μm in other experiments. The concentration of cytochrome *c* or lysozyme hydrochloride was 10 μg·mL^−1^ in all experiments. The concentration of other all compounds was 100 μm in all experiments.

Through this screening procedure, we also found five compounds that enhanced the binding of Aβ oligomers to EphB2 to more than 200% (Table 2).

**Table 2:** Compounds that enhance the binding of Aβ oligomers to EphB2

Compound	Aβ binding (% of control)
Domiphen bromide	386.8
Dopamine hydrochloride	262.8
Pentamidine isethionate	238.6
Levodopa	214.9
Fluvoxamine maleate	206.6

Five compounds that increased the Aβ oligomer binding to EphB2 to more than 200% in the screening are listed. Values are mean (*n* = 2). The concentration of drugs was 100 μm.

**Figure 2:**
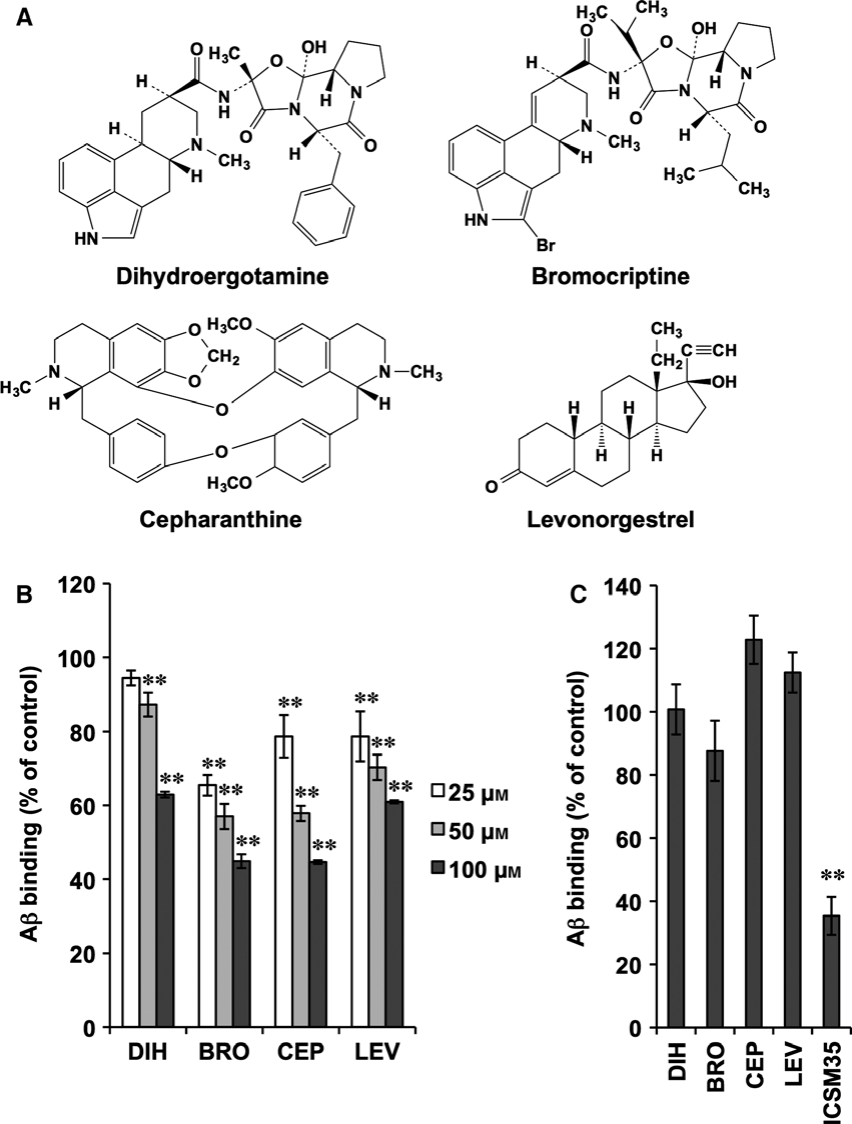
Selective inhibition of the binding of Aβ oligomer to EphB2 by selected compounds. (A) Structures of the four selected compounds are shown. (B) The indicated concentration of each compound was added simultaneously with Aβ oligomers to EphB2‐coated wells, and the amount of bound biotin‐Aβ was determined. (C) Each compound (100 μm) or ICSM35 (2 μg·mL^−1^) was added simultaneously with Aβ oligomer to PrP^C^‐coated wells, and the amount of bound biotin‐Aβ was determined. Values represent the mean ± SD (*n* = 3). **P < 0.01. Basically similar results were obtained in another independent experiment.

To select compounds that selectively inhibited the Aβ oligomer binding to EphB2, we next examined the effects of these 22 compounds on the binding of Aβ oligomers to PrP^C^. We found four compounds (DIH, BRO, CEP, and LEV; Fig. 2A) that inhibited less than 30% of the Aβ oligomer binding to PrP^C^ (Table 1). As shown in Fig. 2B, each of these four compounds inhibited the binding of Aβ oligomers to EphB2 in a concentration‐dependent manner. Additionally, under conditions in which an antibody against PrP^C^ (ICSM35) 7 inhibited the binding of Aβ oligomers to PrP^C^, none of these four compounds inhibited the binding (Fig. 2C). These results suggested that these four compounds selectively inhibited the binding of Aβ oligomers to EphB2.

### Effects of selected compounds on the binding of ephrinB2 to EphB2

**Figure 3:**
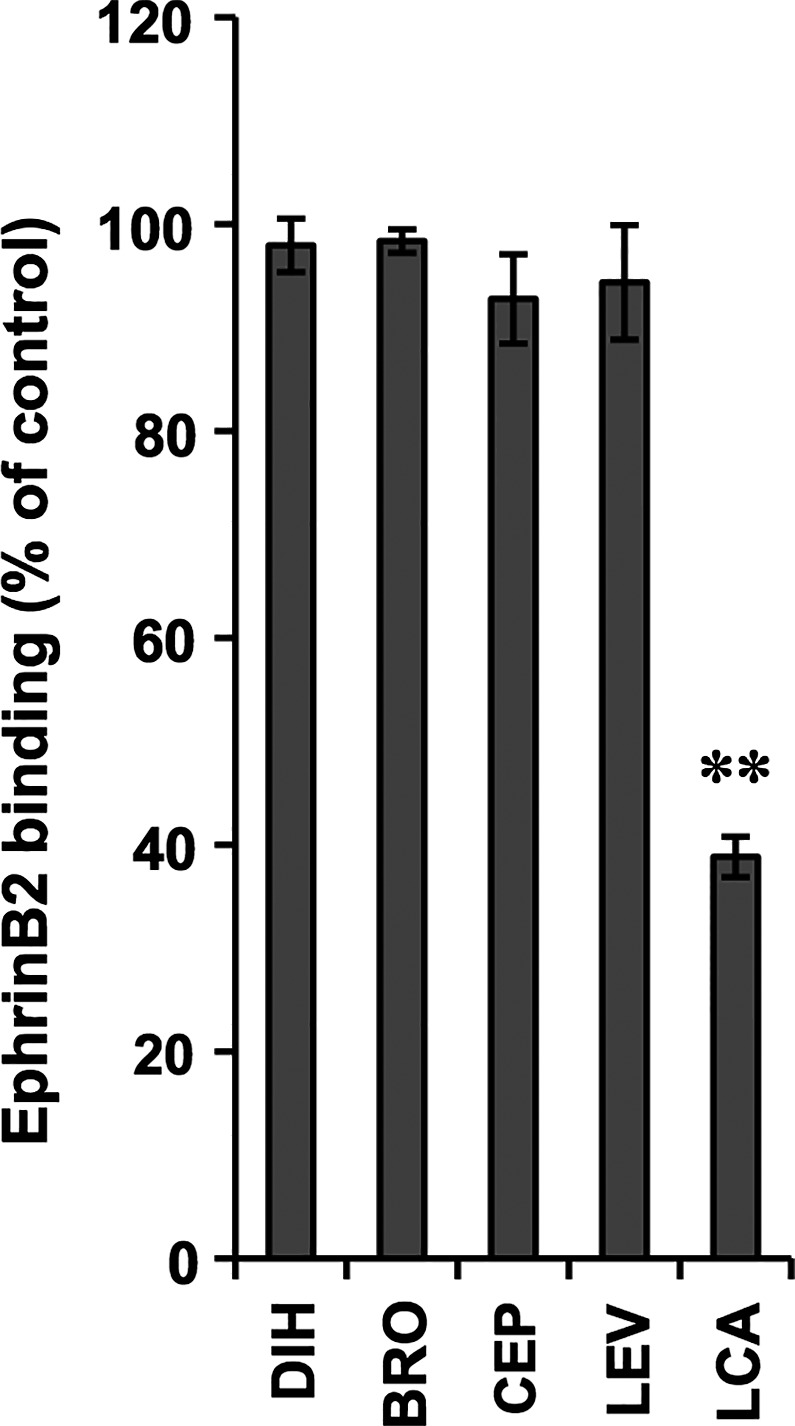
Effects of selected compounds on the binding of ephrinB2 to EphB2. Each compound (100 μm) was added simultaneously with biotinylated ephrinB2‐Fc to EphB2‐coated wells, and the amount of bound biotinylated ephrinB2‐Fc was determined. Values represent the mean ± SD (*n* = 3). **P < 0.01. LCA, lithocholic acid. Basically similar results were obtained in another independent experiment.

The results in Fig. 2 suggested that the four selected compounds interacted with EphB2 rather than with Aβ oligomers to inhibit their binding. Thus, it is possible that these compounds may inhibit the binding between EphB2 and its endogenous ligands, which may cause adverse clinical effects. Thus, we examined the effects of these four compounds on the binding of one of the endogenous ligands, ephrinB2, to EphB2. As shown in Fig. 3, none of these four compounds inhibited the binding of ephrinB2 to EphB2. We confirmed the inhibitory effect of lithocholic acid, which interferes with Eph–ephrin interactions 18, on the binding of ephrinB2 to EphB2 (Fig. 3).

Recently, some compounds were reported to inhibit the binding of Aβ to its receptors. For example, Deane *et al*. 19 identified FPS‐ZM1 as an inhibitor for the binding of Aβ to the receptor for advanced glycation end products, and Risse *et al*. identified Chicago Sky Blue 6B as an inhibitor for the binding of Aβ to PrP^C^ 20. Furthermore, Fu *et al*. reported that rhynchophylline binds to EphA4 and stimulated LTP in the presence of Aβ oligomer 21.

As described above, Aβ oligomers directly bind to EphB2 and induce its degradation, resulting in LTP impairment and memory dysfunction 5. Because artificial induction of EphB2 expression in the dentate gyrus rescues LTP and memory function in a mouse model of AD 5, induction of EphB2 expression is a potential drug target for treatment of AD. However, EphB2 is also involved in the development of cancer 22, 23 and may stimulate cancer progression. Thus, inhibiting the binding between Aβ oligomers and EphB2 is a better target for developing drugs aimed at AD treatment. Here, we found 22 compounds with such activity. Among them, 18 compounds also inhibited the binding of Aβ oligomers to PrP^C^, whereas four compounds did not. Although we focused on the latter group for development of AD therapeutics, the former group is also interesting. In addition to EphB2, other receptors for Aβ oligomers, such as PrP^C^ 6, 7, 24, leukocyte immunoglobulin‐like receptor B2 25, and IgG Fcγ receptor II‐b 26, are involved in the inhibitory effect on LTP and in neurotoxicity. Therefore, compounds that inhibit the binding of Aβ oligomers to various receptors may have beneficial effects for treatment of patients with AD. Indeed, among the 18 compounds identified here, montelukast 27, minocycline 28, 29, and bexarotene 30 reportedly have beneficial effects in animal models of AD.

The four compounds we identified that selectively inhibited the binding of Aβ oligomers to EphB2 (DIH, BRO, CEP, and LEV) are approved for use in humans as treatments for migraine, Parkinson's disease, and leucopenia, as well as for use as a contraceptive, respectively. Among these compounds, DIH, BRO, and CEP penetrate the blood–brain barrier 31, 32, 33. Thus, our results suggest that these compounds, especially DIH, BRO, and CEP, may be safe and effective drugs for treatment of AD. On the other hand, these compounds required relatively high concentration to show their inhibitory effect on the binding of Aβ oligomers to EphB2. Therefore, we also consider the drug modification, using these approved medicines as leads.

As described above, the compounds identified in this study are expected to ameliorate Aβ oligomer‐induced LTP impairment. EphB2 overexpression suppresses Aβ oligomer‐induced neurotoxicity in cultured hippocampal neurons 34, and activation of EphB2 attenuates tau phosphorylation in a mouse model of AD 35. Because both Aβ oligomer‐induced neurotoxicity and tau phosphorylation are important factors in AD pathology, the four compounds identified here may also prove useful for AD treatment through these mechanisms.

In conclusion, we screened for compounds that selectively inhibit the binding of Aβ oligomers to EphB2 from a library of approved drugs already in clinical use, and identified dihydroergotamine mesilate, bromocriptine mesilate, cepharanthine, and levonorgestrel. We propose further analysis of these compounds as candidates for AD treatment.

## Author contribution

KS, TI, and TM conceived and designed the project; KS and TA acquired the data; KS analyzed and interpreted the data; and KS, TI, and TM wrote the paper.
